# Diversifying Isoprenoid Platforms *via* Atypical Carbon Substrates and Non-model Microorganisms

**DOI:** 10.3389/fmicb.2021.791089

**Published:** 2021-12-02

**Authors:** David N. Carruthers, Taek Soon Lee

**Affiliations:** ^1^Joint BioEnergy Institute, Emeryville, CA, United States; ^2^Biological Systems and Engineering Division, Lawrence Berkeley National Laboratory, Berkeley, CA, United States

**Keywords:** isoprenoids, metabolic engineering, synthetic biology, non-model organisms, C1 metabolism, terpenes

## Abstract

Isoprenoid compounds are biologically ubiquitous, and their characteristic modularity has afforded products ranging from pharmaceuticals to biofuels. Isoprenoid production has been largely successful in *Escherichia coli* and *Saccharomyces cerevisiae* with metabolic engineering of the mevalonate (MVA) and methylerythritol phosphate (MEP) pathways coupled with the expression of heterologous terpene synthases. Yet conventional microbial chassis pose several major obstacles to successful commercialization including the affordability of sugar substrates at scale, precursor flux limitations, and intermediate feedback-inhibition. Now, recent studies have challenged typical isoprenoid paradigms by expanding the boundaries of terpene biosynthesis and using non-model organisms including those capable of metabolizing atypical C1 substrates. Conversely, investigations of non-model organisms have historically informed optimization in conventional microbes by tuning heterologous gene expression. Here, we review advances in isoprenoid biosynthesis with specific focus on the synergy between model and non-model organisms that may elevate the commercial viability of isoprenoid platforms by addressing the dichotomy between high titer production and inexpensive substrates.

## Introduction

Isoprenoids are ubiquitous across all domains of life and span a wide and varied range of natural products. Isoprenoids are characterized by condensation of the five carbon precursor molecules isopentenyl diphosphate (IPP) and dimethylallyl diphosphate (DMAPP), which are typically generated through either the mevalonate (MVA) or methylerythritol phosphate (MEP) pathways. The ease with which specialized synthases and cytochromes can conjugate or decorate these precursors has led to a uniquely diverse class of chemicals. Estimates of natural isoprenoid compounds in the last several decades have steadily increased from 20,000 (Chappell, 1995) to over 70,000 (Moser and Pichler, 2019). The advent of advanced sequencing, -omics, and bioinformatics technologies coupled with protein structural software and flux balance analyses have facilitated a veritable revolution in synthetic biology and assured the continued elucidation of isoprenoid compounds through bioprospecting and biosynthetic efforts.

Isoprenoids serve a number of critical roles both as primary and secondary metabolites. Primary metabolites are essential to cell survival and propagation. They include carotenoids that serve as auxiliary molecules for photoprotection and antioxidants (carotene, lycopene, lutein, and zeaxanthin) as well as sterols that help maintain membrane structure. Other isoprenoids function as components of dolichols, quinones, and essential proteins that aid in glycosylation and electron transport (Chappell, 1995). Secondary isoprenoid metabolites impart a non-essential benefit to cells usually by providing some defensive benefit or, in higher plants, hormone signaling. As for isoprenoids, these include pigments, fragrances, essential oils, and defensive chemicals that are most prominent in higher plants. Many secondary metabolites have attracted particular interest due to their applications as pharmaceuticals [e.g., artemisinin (Ro et al., 2006) and pacilitaxel (Biggs et al., 2016)], nutraceuticals, biofuels [e.g., isoprenol (Kang et al., 2019), prenol (Zheng et al., 2013), bisabolene, and limonene (Alonso-Gutierrez et al., 2013)], and cosmetics (Schempp et al., 2018). Hybrid technologies have capitalized on isoprenoid versatility through semi-synthetic approaches to generate elastomers (Della Monica and Kleij, 2020). Collectively, the bioproduction of these chemicals has enabled access to multibillion dollar chemical markets.

Microbial pathway engineering has proven especially successful in *Escherichia coli* and *Saccharomyces cerevisiae*, which have produced many of the aforementioned isoprenoid compounds. *E. coli* and *S. cerevisiae* maintain certain metabolic advantages including a fast growth phenotype, historical breadth of knowledge, ease of transformation and hence heterologous protein expression, substrate specificity, and published successes of bioproduction (Vickers et al., 2017; Ward et al., 2018). These advantages are complemented by specialized synthetic biology strategies that enable tuning of ribosome binding site and promoter strength, codon optimization of heterologous proteins, protein fusions, and the knocking out of competing pathways. In recent years, this has been accomplished by systematic gene downregulation using regulatable CRISPR interference systems (Kim et al., 2016; Tian et al., 2019) that express a modified dCas9 protein for fine-tuning of the overall pathway and optimization of target production. Furthermore, *E. coli* endogenously generates isoprenoids through the MEP pathway while *S. cerevisiae* utilizes its native MVA pathway, together enabling researchers to combine synthetic biological toolkits with the abundance of information of these strains to facilitate high-titer isoprenoid production. As for downstream isoprenoid functionalization, these metabolic chassis are genetically tractable whereas many natural isoprenoid production pathways are prevalent in recalcitrant organisms that make high-titer production infeasible. Only recently have certain non-model organisms been engineered to yield comparable or higher isoprenoid titers than in *E. coli* and *S. cerevisiae*.

Despite the clear successes of isoprenoid production, *E. coli* and *S. cerevisiae* have significant disadvantages that limit successful bioproduction at scale. Precursor limitations, either the availability of IPP and DMAPP for direct synthesis of isoprenoids or the availability of MVA/MEP precursors, have been identified as a major obstacle to advancing isoprenoid synthesis (Zu et al., 2020). Fine-tuning of metabolic pathways within the cell to balance cofactor supply by downregulation or upregulation of select enzymes has been identified as a major engineering opportunity and, although generally successful, often involves strain-specific and product-guided strategies (Zu et al., 2020). Scaling of successful production is also limited by the necessity of episomal expression systems, which are ill-suited for industrial production due to the necessity of selective markers and their general instability.

A second major challenge in industrial isoprenoid biosynthesis is simultaneously increasing titer, rate, and yield while reducing the environmental and monetary burden intrinsic to industrial production. Precursor limitations are also complicated by isoprenoid production platforms that rely on sugar-based metabolism. Although sugars like glucose and glycerol provide high MVA/MEP flux by generation of G3P/pyruvate or acetyl-CoA, respectively, the high production costs are prohibitive to competition with petroleum-derived analogs. The greatest cost drivers of isoprenoid biosynthesis stem from carbon feed, which accounts for over 90% of production costs, and product yield (Wu and Maravelias, 2018).

A promising solution to exorbitant substrate costs is carbon source switching, especially to carbon waste streams like cellulosic biomass or C1 substrates (e.g., methane, methanol, carbon dioxide, and formate). Recent estimates assert that sugar switching from glucose to pretreated cellulosic biomass could yield a 53% decrease in cost (Wu and Maravelias, 2018) with further gains if organisms can simultaneously consume multiple substrates (e.g., hexose and pentose sugars). Growth and production on atypical carbon sources and native generation of secondary metabolites is prevalent among many microorganisms. Recent advances have capitalized on the diversity of microbial carbon assimilation pathways, especially in the elucidation of synthetic and natural C1 metabolic pathways that enable access to cheap, abundant carbon sources (Aldridge et al., 2021).

Many archaea have also evolved a robust array of resistance strategies to cope with inhibitory chemicals and conditions. These include tolerance mechanisms (efflux pumps, heat tolerance, membrane modifications, and general stress resistance; Dunlop, 2011) that facilitate extremophilic growth in inhospitable environments like anaerobic conditions or deep sea vents. These mechanisms in some cases directly involve secondary metabolite production and even enable enhanced tolerance to secondary metabolite toxicity (Dunlop et al., 2011). Toxicity tolerance is an appealing phenotype for biofuel production systems as well as for survival on substrates that are typically toxic to many microorganisms like pretreated lignocellulosic biomass (Dunlop et al., 2011). To date, bioproduction on pretreated cellulosic biomass has proven challenging due to the associated toxicity of the substrate, especially the prevalence of aromatic compounds. In response, researchers have begun focusing on resilient bioproduction chassis like *Pseudomonas putida* and *Rhodosporidium toruloides* that can readily degrade aromatic compounds (Yaegashi et al., 2017; Johnson et al., 2019).

Conversely, microbes with unique phenotypes tend to have limited metabolic toolkits available. Next generation sequencing technologies have expedited exploration and characterization of novel organisms from unique environments, yet direct engineering of such organisms for production remains a fundamental challenge. Neither *E. coli* or *S. cerevisiae* naturally accumulate isoprenoids at high titer and bioproduction is often limited to heavily modified strains with inducible episomal expression systems. Even so, the highest production of isoprenoids has been achieved in *E. coli* and *S. cerevisiae* (Moser and Pichler, 2019). As a result, there is a significant disparity between model and non-model isoprenoid production.

Addressing the disparity between the prevalence of nature’s clever solutions to environmental challenges and the genetic tractability of those organisms remains a principal obstacle in isoprenoid bioproduction. In many instances, it poses the question of whether to heterologously express pathways in common metabolic chassis or to optimize pathways *in situ*, both of which come with drawbacks. In this review, we highlight recent advances in core understanding of isoprenoid synthesis, namely the elucidation of the archaeal MVA pathways, precursor flux modulation, and how those discoveries have contributed to novel isoprenoid production schemes. We then explore the exchange between lessons learned in the metabolic engineering of *E. coli* and *S. cerevisiae* and of non-model microorganisms with natural predispositions for atypical and economical carbon substrates. Pathways include C1 metabolism in methylotrophic organisms (*Methanosarcina* sp., *Methylorubrum extorquens*) and phototrophic microbes (cyanobacteria, purple non-sulfur bacteria, diatoms, and green algae) capable of fixing CO_2_. We also explore advances in engineering of oleaginous yeast naturally capable of efficient lipid toleration and accumulation (*R. toruloides* and *Y. lipolytica*) and finally soil bacteria with special focus on their propensity for survival on and degradation of aromatic substrates (*B. subtilis* and *P. putida*). Collectively, these advances move isoprenoid biosynthesis toward economic and environmental feasibility.

## Advances in Isoprenoid Pathway Construction:

**Figure 1:**
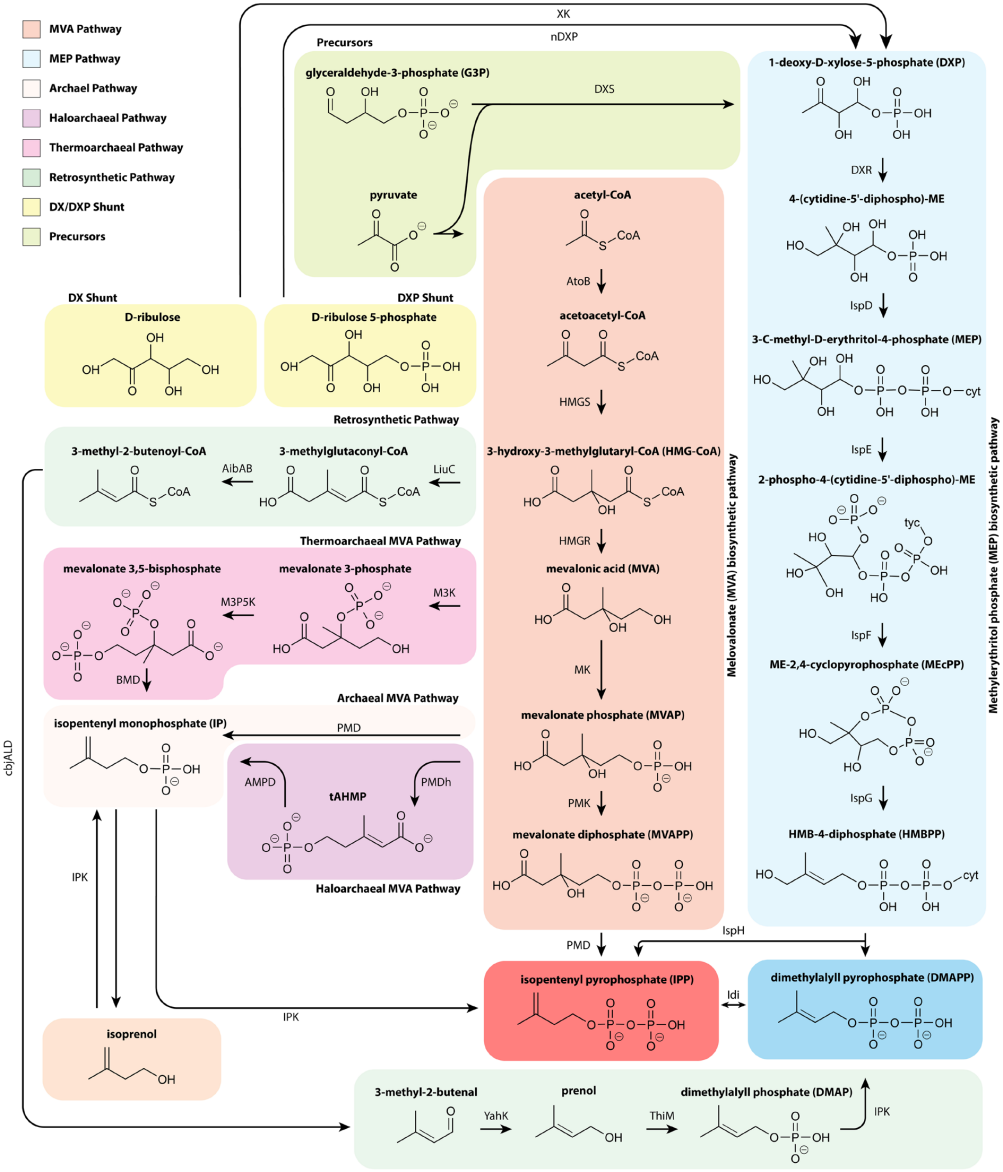
A depiction of isoprenoid synthesis through the core 6 enzyme MVA and 7 enzyme MEP pathways. Also depicted are the newly discovered archaeal branches from the MVA pathway. The thermoarchaeal-type branch begins with mevalonic acid whereas the archaeal and haloarchaeal-type branches stem from MVAP. Typically, isoprenoids are synthesized by acetyl-CoA, pyruvate, and G3P, however IPP and DMAPP can also be synthesized from C5 alcohols, D-ribulose or D-ribulose-5-phosphate, and a synthetic route in which HMG-CoA is ultimately converted to prenol. AibAB, 3-methylglutaconyl-coenzyme decarboxylase; AtoB, acetyl-CoA acetyltransferase; BMD, bisphosphomevalonate decarboxylase; cbjALD, 3-methylcrotonyl-CoA reductase; DXR, 1-deoxy-D-xylulose 5-phosphate reductase; DXS, 1-deoxy-D-xylulose 5-phosphate synthase; HMGR, 3-hydroxy-3-methylglutaryl-CoA reductase; HMGS, 3-hydroxy-3-methylglutaryl-CoA synthase; IDI, isopentenyl diphosphate isomerase; IPK, isopentenyl phosphate kinase; IspD, 2-C-methyl-D-erythritol 4-phosphate cytidylyltransferase; IspE, 4-diphosphocytidyl-2-C-methyl-D-erythritol kinase; IspF, 2-C-methyl-D-erythritol 2,4-cyclodiphosphate synthase; IspG, 4-hydroxy-3-methylbut-2-enyl diphosphate synthase; IspH, 4-hydroxy-3-methylbut-2-enyl diphosphate reductase; LiuC, 3-hydroxy-3-methylglutaryl CoA dehydratase; M3K, Mevalonate 3-kinase; M3P5K, Mevalonate 2-phosphate-kinase; nDXP, 1-deoxyxylulose-5-phosphate synthase; PMD, phosphomevalonate decarboxylase; PMK, phosphomevalonate kinase; tAHMP, anhydromevalonate diphosphate decarboxylase; ThiM, hydroxyethylthiazole kinase; XK, xylulose kinase; YahK, aldehyde reductase.

All isoprenoids are generated from the common cellular precursors acetyl-CoA or glyceraldehyde 3-phosphate (G3P) and pyruvate *via* either the MVA or MEP pathway, respectively (Figure 1). These pathways share no homology and are evolutionarily distinct. Comparisons of the MVA and MEP pathway efficiencies, cofactors, and energetic requirements have been well documented in previous reviews (Dugar and Stephanopoulos, 2011; Yadav et al., 2012). The recent characterization of archaeal MVA pathways, shunts, and alternative precursors for these pathways have harbored the development of unique and more efficient routes for isoprenoid production (Kang et al., 2017; Hayakawa et al., 2018; Clomburg et al., 2019).

### Mevalonate Pathway

The MVA pathway is native to eukaryotes, some ancient and often predatory gram-positive bacteria (Pasternak et al., 2013), as well as, with some significant deviations, archaea (Boucher et al., 2004). The canonical MVA pathway commences with a Claisen condensation of two acetyl-CoA thioester molecules followed by five sequential enzymatic steps that ultimately yield IPP. IPP is then converted to DMAPP by the isopentenyl diphosphate isomerase (IDI) for further condensation into isoprenoid compounds.

Over the last decade, the origin of the archaeal MVA pathway – either progenating from horizontal gene transfer or a cenancestor – has been hotly debated. However, recent analysis of monophylogenetic candidate phyla radiation and DPANN (Diapherotrites, Parvarcheota, Aenigmarchaeota, Nanoarchaeota, and Nanohaloarchaeota) have provided conclusive evidence to support an extant ancestral MVA in all domains of life (Castelle and Banfield, 2018). Most notably, the archaeal pathway lacks PMK, PMD, and IDI1. Instead, archaea have an alternative IDI2 similar in function to IDI1 and rely upon the recently discovered isopentenyl phosphate kinase (Dellas et al., 2013) to generate IPP through unique MVA intermediates. Specifically, three distinctive archaeal MVA pathways have been elucidated: the haloarchaea-type MVA, the thermoplasma-type MVA, and the archaeal MVA pathway that is conserved throughout the kingdom (Hayakawa et al., 2018; Thomas et al., 2019; Yoshida et al., 2020) as depicted in Figure 1.

Beyond the perplexities of phylogenetic classification (hereditary, horizontal gene transfer, etc), the elucidation of archaeal MVA pathways and their associated enzymes has proven instrumental in optimizing *S. cerevisiae/E. coli* production titers by capitalizing upon enzyme promiscuity or efficiency. Collectively, heterologous expression and fine-tuning of the MVA pathway to minimize flux bottlenecks has included the expression of genes across different domains. Overexpression of HMGS and HMGR from *Staphylococcus aureus* (Tsuruta et al., 2009) as well as a kinase from the archaeal *M. mazei* (Primak et al., 2011), for example, was successfully shown to improve C5 isoprenoid accumulation and laid the groundwork for longer chain isoprenoid production *via* the MVA pathway (George et al., 2015).

### Methylerythritol Phosphate Pathway

The MEP pathway is native to most Gram-negative bacteria and cyanobacteria as well as to algae and higher plants, but in the latter eukaryotes it is compartmentalized in the plastid. Despite being theoretically more efficient than the MVA pathway, the MEP pathway is more tightly regulated and challenging to engineer. Studies have elucidated rate limiting enzymatic steps in the MEP pathway, namely IDI and DXS, for β-carotene production (Yuan et al., 2006). However, overexpression of MEP pathway genes can also have deleterious effects on actual isoprenoid synthesis due to accumulation of intermediates. Our fundamental understanding of MEP pathway regulation is incomplete, encompassing some feedback and feedforward mechanisms between downstream isoprenoids and MEP intermediates (Bitok and Meyers, 2012). Studies in higher plants and algae, which have demonstrated that circadian light/dark cycling have a significant influence on pathway regulation, further complicate our understanding (Vranová et al., 2012).

A recent metabolic control analysis employed -omics studies with recombineering to show that, normalized to DXS flux, IspG is the rate limiting step for isoprene synthesis with other enzymes increasing linearly with DXS concentration (Volke et al., 2019). This is an important finding as isoprene is the simplest hemiterpene and therefore a good reporter for MEP tuning. Yet production did not increase with overexpression of IspG and IspH, suggesting instead that other cofactors may be limiting (Volke et al., 2019). Indeed, careful balancing of IspG and IspH expression has shown enhanced β-carotene and α-lycopene production (Li et al., 2017), suggesting that pathway tuning should be based on an intricate, product-driven approach (e.g., different tuning for isoprene vs. higher chain length terpenoids) rather than an intuitive, generalizable rule. In general, careful expression balancing has been the most successful strategy to MEP pathway optimization due to the complexity of regulatory mechanisms in *E. coli*, though even careful balancing in other organisms like the cyanobacterium *Synechococcus elongatus* has proven challenging (Englund et al., 2018).

### Synthetic Isoprenoid Production Pathways

Although essential to isoprenoid production, high IPP and DMAPP accumulation is toxic and can result in significant growth inhibition (George et al., 2018). This dilemma has led to a number of clever strategies for synthetic “growth-decoupled” and “bypass” isoprenoid production routes that comprise components of MVA/MEP pathways but avoid IPP/DMAPP accumulation. Many of these strategies have been informed or directly use elements from the recently elucidated archaeal MVA pathways, either through direct codon optimized expression or as templates for engineering promiscuous activity. A mevalonate decarboxylase from *Halobacterium volcanii*, for example, was expected to demonstrate conversion of MVAP to IP and employed as a template to rationally design PMDs for C5 alcohol production. The strategy successfully enhanced isoprenol production by bypassing intracellular IPP accumulation (Vannice et al., 2014; Kang et al., 2016). Further mutagenesis of a *S. cerevisiae* PMD in tandem with an endogenous phosphokinase resulted in an IPP-bypass pathway that yields IP and ultimately the highest isoprenol titer reported at 10.8g/L (Kang et al., 2017, 2019).

Retro-biosynthetic approaches postulated that archaeal IPKs could enable phosphorylation of the C5 alcohols (isoprenol and prenol) into IPP and DMAPP, respectively. Direct feeding of alcohols for production of isoprenoid precursors could thereby decouple isoprenoid production from central carbon metabolism. Of particular interest were IPKs from *Halobacterium volcanii*, *Methanothermobacter thermautotrophicus*, *Thermoplasma acidophilum*, and *Methanocaldococcus jannaschii* (Chatzivasileiou et al., 2019). In one study, IPKs from the latter three archaea were screened for activity and cloned into an *E. coli* strain harboring a β-carotene production pathway (Liu et al., 2016). Expression of the IPK from *T. acidophilum* and feeding of 2mM prenol resulted in a 45% increase in β-carotene production and was further improved by site-specific mutagenesis to 97% (Liu et al., 2016). Growth-decoupled production of lycopene was also demonstrated by overexpressing a codon-optimized *T. acidophilum* IPK paired with an endogenous *E. coli* phosphatase PhoN, with titers nearing 190mg/L in an mixture of 2.5mM prenol and isoprenol (Chatzivasileiou et al., 2019; Clomburg et al., 2019; Lund et al., 2019). IPK-mediated production of carotenoid and neurosporene was also improved in *E. coli* by 18-fold and 45-fold, respectively, by decoupling terpene synthesis from central carbon metabolism through production on C5 alcohols (Rico et al., 2019).

Several other strategies utilize two upper MVA pathway genes (*E. coli* AtoB, *Staphylococcus aureus* HMGS) prior to diverging with the expression of the hydratase LiuC and *Myxococcus xanthus* decarboxylase AibAB (Clomburg et al., 2019; Eiben et al., 2020). From there, Eiben et al. demonstrated 80mg/L isopentanol production through subsequent expression of *M. xanthus* AibC and *Clostridium acetobutylicum* AdhE2 (Eiben et al., 2020). In a more holistic approach, AibAB was followed with expression of the *Clostridium beijerinckii* acyl-CoA reductase (cbjALD), and *E. coli* YahK to generate prenol at the highest titer reported for biological production (Figure 1; Clomburg et al., 2019). Conversion of prenol to DMAP was then accomplished by the *E. coli* hydroxyethylthiazole kinase (ThiM) and finally converted to DMAPP by the *M. thermoautotrophicus* IPK (Clomburg et al., 2019). The unique approach employed by Clomburg et al. succinctly demonstrates how novel enzymes wrought by recent discoveries can be instrumental in designing pathways that circumvent metabolic bottlenecks to yield high titer production platforms.

To conclude, several studies have explored novel precursors to the MEP pathway using ribulose. The initial step in the MEP pathway, condensation of G3P and pyruvate with DXS, results in production of DXP with the loss of a CO_2_ molecule or one sixth of total carbon (Kirby et al., 2015). A D-ribulose 5-phosphate shunt by nDXP was initially explored in *E. coli* by a semi-rational approach, which identified *yajO* and *ribB* gene mutants as candidate enzymes and improved carbon efficiency by direct conversion of C5 sugars to C5 MEP intermediates. Expression of the nDXP shunt enabled a 4-fold increase in MEP derived bisabolene production (Kirby et al., 2015). This approach was further demonstrated in *P. putida* by expression of the mutant *ribB* gene, but with low efficiency (Hernandez-Arranz et al., 2019). In an analogous work, promiscuous activity of fructose-6-phosphate aldolase in *E. coli* was used to generate D-ribulose from the glycolaldehyde and hydroxyacetone. Another DXP shunt overexpressed a native xylulose kinase (King et al., 2017). These novel shunts, like the archaeal informed MVA pathways, have the potential to alleviate precursor flux limitations. While the MVA pathway modifications have had clear success, it has yet to be determined whether these shunts can address the regulatory challenges associated with MEP derived isoprenoid production.

## Advances in Isoprenoid Functionalization

The C5 precursors IPP and DMAPP are dephosphorylated, cyclized, and modified to create a structurally diverse group of over 70,000 chemicals through a coordinated enzyme network (Kirby and Keasling, 2009; Moser and Pichler, 2019). The first stage or module of isoprenoid biosynthesis is characterized by the successive addition of the diphosphate precursor *via* head-to-head or tail-to-head condensation. The second module is an operation or series of operations conducted by terpene synthases (TSs) in which the terpenoid skeleton is dephosphorylated and cyclized. The third module involves further decoration by cytochrome P450s (CYPs), acetyltransferases, methyltransferases, dehydrogenases, and in some cases, glycosylations. This overall framework is consistently repeated in nature with some variations (Zhou and Pichersky, 2020). In this section we discuss recent advances in the functionalization of isoprenoids. Broad ranges of chemical production targets have been demonstrated and scaled from biofuels to pharmaceuticals by heterologous expression of prenyl diphosphate synthases, TSs, and CYPs.

### Cytochrome P450s

Heme-thiolate monooxygenases or CYPs are an interesting class of enzymes that functionalize terpenes through oxygenation reactions (hydroxylation, dealkylation, demethylation, decarboxylation, cyclization, C-C bond cleavage, among others) and present an important opportunity for generating highly decorated natural products. To date, over 300,000 CYPs have been discovered, with less than 1% actually characterized (Li et al., 2020; Liu et al., 2020). There is particular interest to produce CYP-derived terpenoids in microbial chassis due to the high barriers of slow growth and costly deconstruction inherent to native plant extraction.

Engineering CYPs has significant implications for novel and unnatural bioproducts (Helfrich et al., 2019; Xiao et al., 2019). The range of oxygenated terpenes is complemented by the sheer expanse of CYP availability in plants (Zhou and Pichersky, 2020). For example, CYPs are critical for the production of bioactive molecules with high pharmacological impacts. Case studies of microbial expression include production of precursor intermediates to artemisinin (CYP71AV1, aaCPR) and taxadiene (CYP725A4, tcCPR), which are natively produced by wormwood (*Artemisia annua*) and the Pacific yew tree (*Taxus brevifolia*) and were heterologously expressed in *E. coli* and *S. cerevisiae*, respectively. Indeed, the diversity and complexity of plant TSs, presented elsewhere (Karunanithi and Zerbe, 2019) offer tremendous potential as candidates for microbial production.

These specific examples demonstrate successful engineering of CYPs for pharmaceutical production, however functional plant CYP expression in microbes has proved challenging. Unfortunately, *E. coli* cannot naturally perform most posttranslational modifications and expression of membrane bound proteins like CYPs generates inclusion bodies or aggregates of insoluble proteins. The production of oxygenated taxanes in *E. coli* (Biggs et al., 2016), for example, required extensive engineering of the CYP redox-partner cytochrome P450 reductase (CPR) pairings, N-terminal modifications for better solubility, and had significant repercussions on upstream MEP pathway balance. These pairings and modifications are necessary for any heterologous CYP expression in *E. coli* and vary depending on the selected proteins. Remarkably, although yeasts are naturally capable of many posttranslational modifications, express native CPRs, and require less N-terminal modification, meta-analyses have shown that *E. coli* studies tend to have higher yield CYPs than *S. cerevisiae* despite the necessity of many more genetic modifications (Hausjell et al., 2018). Many CYP-reductase pairings have been explored in *E. coli* and *S. cerevisiae* as listed in Table 1.

**Table 1:** CYP expression, reductase pairing, and production of oxygenated terpenoids in various microbial hosts.

CYP	Reductase pair	Source organism(s)	Expression organism	Precursor	Product	Titer	Ref.
CYP71AV1	aaCPR	*Artemisia annua*	*S. cerevisiae*	Amorphadiene	Artemisinic acid	100mg/L	Ro et al., 2006
CYP71AV1	aaCPR	*A. annua*	*E. coli*	Amorphadiene	Artemisinic acid	5.8mg/L	Chang et al., 2007
CYP706B1	ctCPR	*Candida tropicalis*	*E. coli*	Cadinene	8-hydroxycadinene	105mg/L	
CYP71BA1	atCPR	*Zingiber zerumbet*, *A. thaliana*	*E. coli*	α-humulene	8-hydroxy-a-humulene	2.972 μg/L	Yu et al., 2011
CYP76AH1	atCPR1	*Salvia miltiorrhiza*, *A. thaliana*	*S. cerevisiae*	Miltiradiene	Ferruginol	10.5mg/L	Guo et al., 2013
CYP153A6	mCPR	*Mycobacterium sp*.	*E. coli*	Limonene	Perillyl alcohol	100mg/L	Alonso-Gutierrez et al., 2013
HPO	atCPR	*Hyoscyamus muticus, A. thaliana*	*P. pastoris*	(+)-valencene	(+)-nootkanone	208mg/L	Wriessnegger et al., 2014
CYP725A4	tcCPR	*Taxus cuspidata*	*E. coli*	Paclitaxel	Oxygenated taxanes	570mg/L	Biggs et al., 2016
CYP726A20	jcCPR1	*Jatropha curcas*	*S. cerevisiae*	Casbene	Oxidized casbanes	~1g/L	Wong et al., 2018
CYP716A12	atCPR	*Callitropsis nootkatensis*, *A. thaliana*	*Y. Lipolytica*	(+)-valencene	(+)-nootkanone	978.2 μg/L	Guo et al., 2018
CYP71BA1	atCPR	*Z. zerumbet; A. thaliana*	*S. cerevisiae*	a-humulene	A-humulene 8-hydroxylase; zerumbone	40mg/L	Zhang et al., 2018
CYP716A12	atCPR	*Medicago truncatula, A. thaliana*	*Y. lipolytica*	Lupeol	Betulinic acid	26.53mg/L	Sun et al., 2018
CYP716A47	pgCPR1	*Panax ginseng*, *A. thaliana*	*S. cerevisiae*	Dammarenediol II	Protopanaxidiol	11.02g/L	Wang et al., 2019
CYP716A12	mtCPR	*M. truncatula*	*Phaeodactylum tricornutum*	Lupeol	Betulinic acid	0.1mg/L	D’Adamo et al., 2019
BcABA1 BcABA2	bcCPR1	Botrytis cinerea	*S. cerevisiae*	FPP	Abscisic acid	4.7mg/L	Otto et al., 2019
CYP72A63	atCPR1, mtCPR2, mtCPR3, guCPR2	*A. thaliana*, *M. truncatula*, *Glycyrrhiza uralensis*	*S. cerevisiae*	11-oxo-b-amyrin	Glycyrrhetol	31.8mg/L	Sun et al., 2020

*Culture conditions, scale, and medium vary significantly. HPO, hyoscyamus muticus premnaspirodiene oxygenase; CPR, cytochrome P450 reductase*.

**Figure 2:**
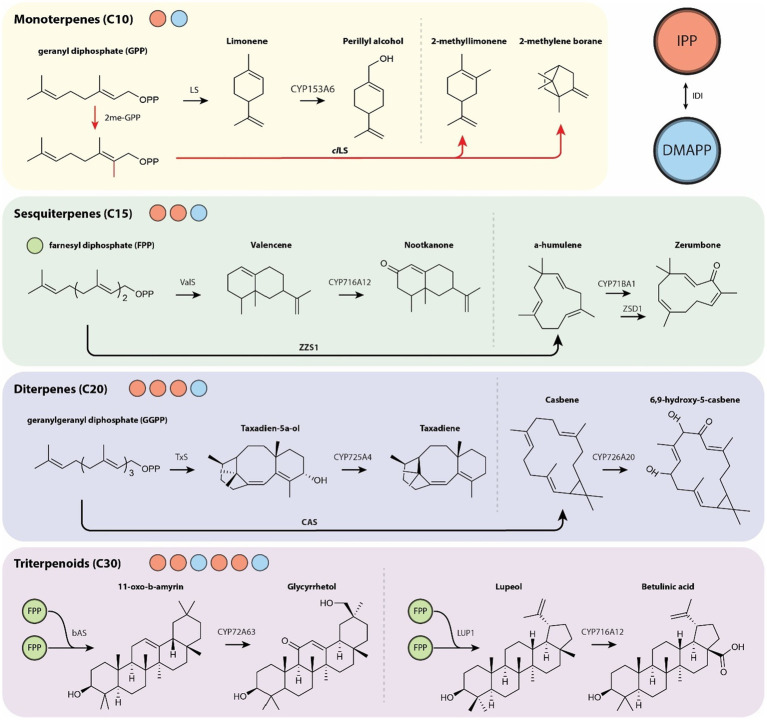
Overview of varied length terpenoids from their diphosphate precursors (red circle, IPP; blue circle, DMAPP; green circle, FPP) with further modification by TSs and subsequent decoration by CYPs. The production of unnatural C11 monoterpenoid compounds *via* methyltransferase 2me-GPP is indicated with red arrows. CAS, casbene synthase; LS, limonene synthase; *cl*LS, *Citrus limon* limonene synthase; 2me-GPP, GPP methyltransferase; TxS, taxadiene synthase; bAS, β-amyrin synthase; LUP1, lupeol synthase 1; ValS, valencene synthase; ZSD1, zerumbone synthase; ZZS1, α-humulene synthase.

In recent years, microbial expression of CYPs has produced many variable length terpenoids (Figure 2). Of special significance are the monoterpene perillyl alcohol, an anti-cancer drug, from limonene (Alonso-Gutierrez et al., 2013) and the sesquiterpenes nootkatone, a pharmaceutical, from valencene (Guo et al., 2018) and zerumbone, an antioxidant, from α-humulene (Zhang et al., 2018). Of further interest are the pharmacologically relevant diterpenes taxadiene and oxygenated casbenes as well as triterpenoids glycerrhetol from 11-oxo-b-amyrin and betulinic acid from lupeol.

There have been many breakthroughs in CYP-derived terpenes in the last 5years that may provide guidance in the engineering of other CYP expression systems. Their interest has led to toolkits for enhanced CYP selection for targeted product engineering to streamline oxyfunctionalization of terpenes (Hernandez-Ortega et al., 2018). Challenges remain with regards to CYP promiscuity, enantiomeric purity, and, perhaps most importantly, production titers. Hopefully, such tools will guide hypotheses and penetrate into the library of known but currently inaccessible plant bioactive terpenes for use in high titer therapeutic production.

### Atypical Terpenoid Production

Several studies have investigated novel isoprenoid chain lengths through the expression of unique S-adenosyl-methionine (SAM)-dependent methyltransferases that methylate the fundamental IPP/DMAPP building blocks and thereby break the natural C5 dogma. In a pioneering study, a GPP methyltransferase (2meGPP) from the cyanobacterium *Pseudanabaena limnetica* was expressed in *S. cerevisiae* with its native 2-methylisoborneol synthase and then with seven distinct plant monoterpene synthases (Ignea et al., 2018). Each synthase generated a unique fingerprint of novel C11 compounds. By demonstrating a range of C11 targets, the authors provided a proof of concept for future enzyme optimization strategies toward specific C11 targets. In another work, IPP SAM-dependent methyltransferases enable conversion of IPP to C6 and C7 prenyl diphosphates with a methyltransferase from *Streptomyces monomycini*, which could then generate C11, C16, and C17 terpenes as well as zeaxanthin-like C41, C42, and C43 compounds depending on methylation (Drummond et al., 2019). A product-driven approach was also able to elucidate a C-methyltransferase that generated sodoferin, an atypical C16 sesquiterpene (von Reuss et al., 2018). The work demonstrated that sodoferin, which is produced naturally and perhaps exclusively by a *Serratia plymuthica*, could be produced in *E. coli* through heterologous expression of the methyltransferase and TS (von Reuss et al., 2018). A final, completely divergent approach utilizes a thiolase from lepidoptera (butterflies and moths) that naturally produces juvenile hormones in the form of C16 methylated diterpenoids (Eiben et al., 2019). Specifically, the thiolase PhaA condenses a propionyl-CoA with an acetyl-CoA as opposed to the standard AtoB of the MVA pathway, which condenses two acetyl-CoA substrates.

It is probable that the control of specific methylation sites decreases with compound size such that targeting specific triterpenoids would remain an obstacle. In the case of C11 targets, site-directed mutagenesis of the monoterpene synthases did enable higher selectivity (Ignea et al., 2018), which is encouraging for future engineering. These unique approaches have expanded the boundaries of isoprenoid synthesis well beyond the C5 rule, though admittedly practical applications of these novel compounds have yet to be realized.

### Meroterpenoids

Partial isoprenoids or meroterpenoids are a class of compounds containing an isoprenoid chain paired with another structure and may have beneficial bioactive properties. Broadly, meroterpenoids include cytokinins, quinones, steroids, and porphyrins like heme A and chlorophyll a. The optimization of heterologous meroterpenoids poses a unique engineering challenge as the isoprenoid compound must be cogenerated with another structure, then converted to the terpenoid by a specified synthase.

A good case study is the production of cannabinoids in *S. cerevisiae*. Cannabinoids are of commercial interest but, like many natural products, suffer from low *in planta* yields. In a recent publication, the production of olivetolic acid from acetyl-CoA was engineered using a six gene pathway (Luo et al., 2019). Prenylation of olivetolic acid using a *Cannabis sativa* prenyltransferase (csPT4) and further heterologous synthases led to *in vivo* production of cannabigerolic acid, Δ9-tetrahydrocannabinolic acid, cannabidiolic acid, Δ9-tetrahydrocannabivarinic acid, and cannabidivarinic acid (Luo et al., 2019). The work not only presents a novel production scheme in *S. cerevisiae* but demonstrates the ease with which transgenic elements can be translated into production chassis.

Another relevant example is the production of prenylated flavonoids, which are derived from cyclic amino acid biosynthesis and can serve as nutraceuticals and medicines. They, again, are found in low natural abundance in plant species like *Sophora flavescens*, a shrub and *Humulus lupulus*, brewing hops (Yang et al., 2015). Production of naringenin in *S. cerevisiae* coupled with overexpression of a plant flavonoid prenyltransferase enabled production of the pharmaceutically relevant 8-prenylnaringenin (Levisson et al., 2019). Although both strategies were limited to yeast, they underline the flexibility of engineering meroterpenoid production in microbes to address commercial needs and provide a promising opportunity for accessing low abundance natural products.

Unique meroterpenoids are also generated in high natural abundance in certain microbes. Archaea differ from bacteria primarily in membrane composition. Archaea produce isoprenoid-derived glycerol lipid ethers (namely ester linked sn-glycerol 3-phosphates rather than ether linked sn-glycerol 1-phosphates) that facilitate growth in unique environments. Full reduction of these long length C20/C40 membrane isoprenoid chains is accomplished by downstream geranylgeranyl reductases (GGR). While they provide an evolutionary advantage for survival in extreme conditions, membrane isoprenoids may also be utilized to generate unsaturated chemicals of interest (Jain et al., 2014). Archaeal lipids, namely archaeol and caldarchaeol, have been identified as potentially valuable for the formation of archaeosomes. Archaeosomes are lipid vesicles composed of archaeal derived lipids and have shown higher physicochemical stability than liposomes, a conventional drug delivery system. As a result, archaeosomes have been singled out as a possible adjuvant and could prove particularly valuable in slow release drug delivery systems (Caforio and Driessen, 2017).

## Industrial Production From C1 Chemical Feedstocks

**Figure 3:**
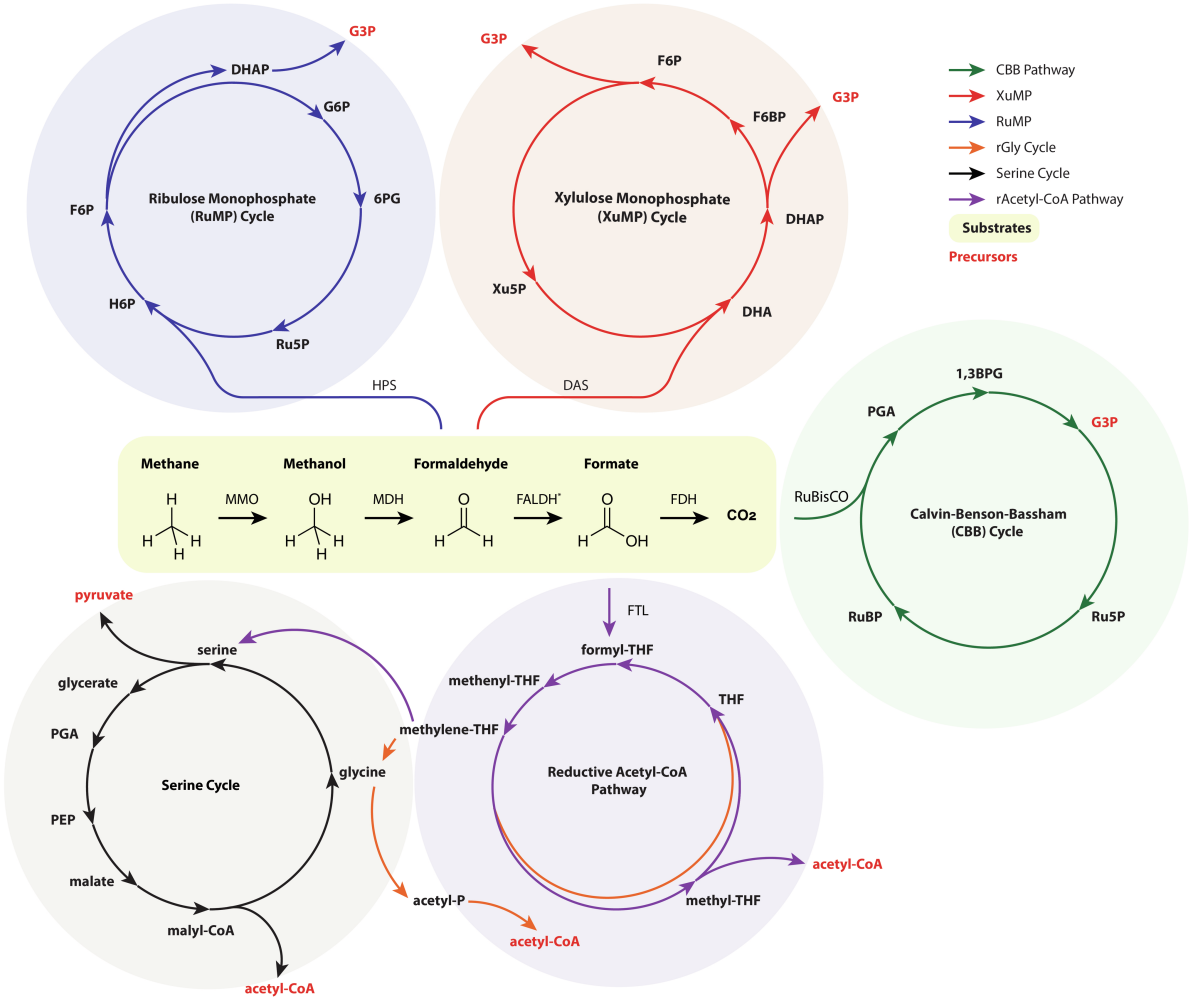
An amalgamated and simplified depiction of relevant C1 metabolic pathways, namely the ribulose monophosphate (RuMP) cycle, the xylulose monophosphate (XuMP) cycle, the Calvin-Benson-Bassham (CBB) cycle, the serine cycle, reductive acetyl-CoA (Wood-Ljungdahl) cycle, and the reductive glycine pathway. Intracycle reactions and conversion of metabolites by glycolysis is not shown. Emphasis is placed on precursors for isoprenoid and central carbon metabolism, namely G3P, acetyl-CoA, and pyruvate. For clarity, only the enzymes involved in the initial C1 assimilation are listed. For simplicity, FALDH is the depicted enzyme for conversion of formaldehyde to formate and the canonical methanogenic reactions are omitted. 1,3BPG, 1,3-bisphosphoglyceric acid; 6PG, 6-phosphogluconate; DAS, dihydroxyacetone synthase; DHA, dihydroxyacetone; DHAP, dihydroxyacetone phosphate; F6BP, fructose 6-bisphosphate; F6P, fructose 6-phosphate; FALDH, formaldehyde dehydrogenase; FDH, formate dehydrogenase; FTL, formate-THF ligase; G3P, glyceraldehyde 3-phosphate; G6P, glucose 6-phosphate; H6P, hexulose 6-phosphate; HPS, hexulose-6-phosphate synthase; MDH, methanol dehydrogenase; MMO, methane monooxygenase; PEP, phosphoenolpyruvate; PGA, 3-phosphoglyceric acid; Ru5P, ribulose 5-phosphate; RuBisCO, ribulose 1,5-bisphosphate carboxylase-oxygenase; RuBP, ribulose bisphosphate; THF, tetrahydrofolate; Xu5P, xylulose 5-phosphate.

C1 substrates are typically generated as industrial and petrochemical byproducts and, in general, C1 substrates are stable, abundant, and inexpensive. Advances in sequestration and hydrogenation of atmospheric CO_2_ *via* heterogeneous catalysts have enabled the conversion of emissions into valuable C_2+_ substrates (Ye et al., 2019). Metabolic engineering of organisms capable of C1 growth is an enticing opportunity for achieving cost parity with petrochemical products while simultaneously improving sustainability metrics. C1 metabolism may be subdivided into phototrophic, methylotrophic, or formatotrophic microbes that consume CO_2_, methane/methanol, and formate/formic acid, respectively. Here, we describe recent approaches for converting C1 substrates into isoprenoid precursors with specific attention to works demonstrating isoprenoid production. The generalized pathways for C1 metabolism are illustrated in Figure 3.

### Methylotrophic Isoprenoid Production

Methane is an abundant byproduct of many chemical processes including fracking and petroleum drilling (Clomburg et al., 2017). In terms of greenhouse gas emissions, methane is approximately 20 times more potent than CO_2_ and excess capacity is flared at refineries, lending to an increase in direct CO_2_ emissions and a loss of revenue (Conrado and Gonzalez, 2014). Methane is also generated through anaerobic digestion of waste biomass. To date, many successful chemical platforms utilize methane as a feedstock to generate alcohols, carboxylic acids, as well as other common C2/C3 chemicals (Kuhl et al., 2012). While biological conversion rates tend to be lower, certain methylotrophic organisms have arisen as potential candidates to capitalize upon methane/methanol availability for more complex bioproduction. In this section, we discuss recent advances in isoprenoid biosynthesis in methylotrophic cell factories across domains.

Archaea are well regarded for their ability to thrive in nutrient-limited anaerobic and extreme conditions. As a result, many archaea have evolved highly efficient strategies for C1 assimilation. On one hand this has helped elucidate more efficient MVA pathways as previously described, but on the other it makes the engineering of tightly regulated archaeal pathways that are geared toward energy conservation thermodynamically challenging. Nonetheless, one study demonstrated production of isoprene from methanol in *Methanosarcina acetivorans* and *Methanosarcina barkeri* under anaerobic conditions and showed a redirection of electron flux from membrane precursors in favor of isoprene accumulation (Aldridge et al., 2021). The diverted isoprene accounted for 4% of total carbon flux.

More substantial success has been achieved in methylotrophic bacteria, many of which thrive in more mesophilic conditions. Methylotrophic bacteria are predominantly divided into two types: Type I assimilates formaldehyde using the RuMP cycle and Type II assimilates formaldehyde *via* the serine cycle. *Methylorubrum extorquens* AM1 (formerly *Methylobacterium extorquens* AM1), a Type II methylotroph, has been studied for over 60years such that many genetic tools are available (-omics data, metabolic networks, genome-scale model; Schrader et al., 2009). *M. extorquens* fermentations have produced methanol-derived products ranging from 1-butanol (Hu and Lidstrom, 2014) to polymers (Orita et al., 2014). A series of stepwise optimizations in *M. extorquens* AM1 included heterologous expression of the *M. xanthus* MVA genes, an FPP synthase from *S. cerevisiae*, α-humulene synthase from *Zingiber zerumbet*, and reduced carotenoid flux. Combinedly, these modifications resulted in the accumulation of 1.65g/L α-humulene on methanol in fed-batch cultivation, which stands as the highest titer reported (Sonntag et al., 2015). Other works have explored high titer production of the MVA pathway intermediates, including 2.59g/L mevalonic acid from methanol on minimal media using a mevalonate biosensor strategy (Liang et al., 2017), which suggests that high titer production of other isoprenoids is also possible. As another attractive feature, *M. extorquens* harbors the ethylmalonyl-CoA pathway (EMCP) that includes a series of anaperlotic activated CoA esters useful for pathway remodeling (Schrader et al., 2009; Schada von Borzyskowski et al., 2018). Interpathway metabolite exchange has risen as a major avenue for further C1 metabolism, especially for formatotrophs as discussed later.

Recently, the methylotrophic yeast *Pichia pastoris* (formerly known as *Komagataella phaffii*) has arisen as a promising candidate for isoprenoid production on methanol. *P. pastoris* maintains several unique characteristics including a tightly regulated and highly expressed alcohol oxidase AOX1, which catalyzes oxidation of methanol to formaldehyde. AOX1 is strongly induced by methanol but repressed by glucose and glycerol (Hartner and Glieder, 2006). As a result, fed-batch production schemes have been designed to partition high cell density growth on glycerol/glucose and production on methanol. This strategy has successfully demonstrated the production of 714mg/L lycopene (Zhang et al., 2020), (+)-ambrein, squalene (Moser et al., 2018), and 208mg/L (+)-nootkatone (Wriessnegger et al., 2014). Interestingly, the latter study successfully used an approach for CYP production that had failed in *S. cerevisiae*. More involved engineering of *P. pastoris* has demonstrated *de novo* production using heterologously expressed Calvin-Benson-Bassham (CBB) Cycle enzymes along with native genes in the xylulose monophosphate (XuMP) cycle and deletion of certain fatty acid enzymes, ultimately yielding a mutant exhibiting autotrophic growth on CO_2_ (Gassler et al., 2020).

Methylotrophic production has garnered special interest for reducing bioproduction costs either through valorization of commercial waste streams or CO_2_ conversion. Table 2 provides a list of methylotrophic production strains, their respective C1 substrates, and product titers. In most cases, titers are significantly lower than comparative production in *E. coli* or *S. cerevisiae* and methylotrophic cultures require significant supplementation with a rich medium that may somewhat reduce the benefits of C1 production.

**Table 2:** Production of isoprenoids by methylotrophic organisms.

Organism	Feedstock	Product	Titer	Ref.
*Methylomonas* sp. 16a	Methane	astaxanthin	2.4mg/g CDW	Ye et al., 2007
*P. pastoris*	Methanol^**^	(+)-nootkanone	208mg/L	Wriessnegger et al., 2014
*P. pastoris*	Methanol^**^	(+)-valencene	136mg/L	
*M. extorquens*	Methanol	a-humulene	1.65g/L	Sonntag et al., 2015
*P. pastoris*	Methanol^**^	(+)-ambrein	105mg/L	Moser et al., 2018
*P. pastoris*	Methanol^**^	squalene	58mg/L	
*P. pastoris*	Methanol^**^	lycopene	714mg/L	Zhang et al., 2020
*M. alcaliphilum*	Methane	α-humulene	0.75mg/g CDW	Nguyen et al., 2020
M. acetivorans	Methanol^*^	isoprene	0.954mM/L	Aldridge et al., 2021
*M. barkeri*	Methanol^*^	isoprene	36.0μM/L	

* *Complex medium*;

** *Carbon sources switched from glycerol to methanol in fed-batch fermentation*.

Past attempts at methylotrophic production have historically been hindered by low carbon and energy efficiencies and addressing these issues by leveraging RuMP/Serine cycles with the CBB cycle have been long postulated (Conrado and Gonzalez, 2014). Recently, major breakthroughs in *P. pastoris* demonstrated *de novo* production using heterologously expressed CBB cycle enzymes, overexpressed native genes in the XuMP cycle, and deletions of certain fatty acid enzymes. The mutant was ultimately capable of autotrophic growth on CO_2_ (Gassler et al., 2020). Likewise, artificial methanotrophy and formatotrophy have been explored in a complex rewiring of *E. coli* (Bennett et al., 2018; Chen et al., 2020; Kim et al., 2020). These seminal works are excellent examples of how systems biology can be applied to tune precursors, adapt strains, and incorporate well-defined isoprenoid pathways for higher production efficiency at lower substrate costs.

### Phototrophic Isoprenoid Production

Phototrophic growth is characterized by the photosynthetic conversion of CO_2_ to sugars *via* complex photoreductive reactions and the Calvin-Benson-Bassham cycle. Photosynthetic organisms naturally produce carotenoids in high concentrations to cope with excess intracellular reactive oxygen species. Specifically, lutein is used in non-photochemical quenching of chlorophyll triplets during photosynthesis (Dall’Osto et al., 2006), zeaxanthin for heat dissipation and photoprotection, while carotene and chlorophyll absorb light. In many cases, gains from the engineering of the genetic architecture of photosynthesis (e.g., light harvesting complexes, RuBisCo) have been limited. Nonetheless, cyanobacteria, which only exhibit the MEP pathway, have become major targets for metabolic engineering due to their genetic plasticity and malleability with respect to isoprenoid precursors, especially through carbon sinks.

Engineered carbon sinks operate on the hypothesis that carbon fixation reactions are faster than downstream carbon utilizing growth reactions such that the accumulation of intracellular carbon metabolites reduces NADPH consumption and ultimately inhibits photosynthesis (Oliver and Atsumi, 2015). Introduction of exogenous genes demonstrated a 1.8-fold increase in carbon yield for the generation of 2,3-butanediol (Oliver and Atsumi, 2015). Others have shown sucrose, ethylene, and isobutyraldehyde production all while enhancing photosynthetic activity through more optimal use of the electron transfer chain (Ducat et al., 2012; Santos-Merino et al., 2021). This effect was also found to be additive when multiple sinks were introduced, suggesting that “sink engineering” could be conceptually applied to secondary metabolite synthesis through downstream modifications of the MEP pathway (Santos-Merino et al., 2021). Several works have capitalized on this upregulation of photosynthesis by combining this source/sink approach with computationally informed modification of limonene synthase, resulting in a 100-fold production improvement in limonene production (Wang et al., 2016) and potential applications for other isoprenoids.

Cyanobacterial studies have also made improvements through direct modification of native isoprenoid pathway genes in combination with a product-specific terminal synthase. *Synechococcus elongatus* and *Synechocystis* sp. PCC 6803 have been primary targets for production with recent attempts focusing on generation of isoprene, with a hallmark study demonstrating 1.26g/L production (Gao et al., 2016; Yang et al., 2016) albeit over several weeks. This feat was accomplished through the overexpression of MEP pathway enzymes though, more importantly, by bioprospecting for a more efficient isoprene synthase. Comprehensive analyses of the MEP pathway metabolic bottlenecks in *S. elongatus* have also been studied by a systematic investigation of each enzymatic step in the MEP pathway, specifically using isoprene as a simple reporter for MEP flux (Englund et al., 2018). The work found that the regulatory circuitry of the *S. elongatus* MEP pathway is, like that of many other MEP pathway harboring organisms, complex and that a simple overexpression of select pathway genes does not necessarily equate to higher/lower production. Despite this complexity, products including squalene (Pattanaik et al., 2020), bisabolene (Sebesta and Peebles, 2020; Rodrigues and Lindberg, 2021), and α-farnesene (Lee et al., 2017) have been produced in *S. elongatus* through some combination of *idi*, *dxs*, *ispA*, and terminal synthase overexpression. It is possible that cyanobacteria could benefit from acetyl-CoA/pyruvate precursor rebalancing. In one study, overexpression of a pyruvate dehydrogenase increased the pool of available acetyl-CoA for isopropanol production (Hirokawa et al., 2020) and could, in theory, be applied to facilitate a heterologously expressed MVA pathway or, in the reverse, to enhance pyruvate accumulation.

Unlike cyanobacteria, eukaryotic algae maintain both the MVA pathway, located in the cytosol, and the MEP pathway, which is sequestered to the chloroplast in proximity to CO_2_-derived metabolites from photosynthesis. Algae have been hailed as candidate bioproduction microbes for many years due to their propagation in many media and thus potential for growth in wastewater streams like agricultural runoff rich in phosphorus and nitrogen. In general, however, eukaryotic algae are notably more challenging to engineer due to their comparably smaller metabolic toolkits and robust regulatory mechanisms on metabolic flux. Studies have established their propensity for some algal isoprenoid production in low titers including patchoulol (Lauersen et al., 2016), bisabolene (Wichmann et al., 2018), and mixed diterpenoids (Lauersen et al., 2018) in the modal alga *Chlamydomonas reinhardtii* with CO_2_ as the sole carbon source. Another alga, *Dunaliella salina*, has been singled out due to its resilience to highly saline environments that serve as a natural antibiotic against contaminants like protozoa, bacteria, dinoflagellates, and other algae. *D. salina* also naturally accumulates β-carotene under abiotic stress and remains one of the few commercially exploited green algae (Borowitzka, 2013; Fachet et al., 2020) along with *Haematococcus pluvialis* for astaxanthin production. Lastly, *Botryococcus braunii*, a colonial green alga, is rich in isoprenoid derived lipids that consist of 35% dry cell weight (DCW) biomass. The isoprenoids generated are characterized by race and consist of either Botryococcenes (C30–C37), methylated squalenes (C31–C34), or odd-number n-alkadienes or trienes (C23–C33; Metzger and Largeau, 2005). Despite their unique composition, broad attempts to culture and optimize isoprenoid production have been limited in part due to slow growth comparative to other green algae (Morales-Sánchez et al., 2017). Somewhat remarkably, both commercial successes stem from unmodified organisms that simply generate isoprenoids under abiotic stress conditions.

Diatoms are a unique subset of algae with a characteristic cell-wall composed of silica. Certain diatoms are capable of generating highly branched isoprenoids (HBIs) like trienes, tetraenes, and pentaenes intrinsic to some diatoms with potential for pharmaceutical or biofuel usage (Athanasakoglou et al., 2019), possibly generated by promiscuous activity of diatom specific farnesyl pyrophosphate synthases (Ferriols et al., 2015). A specific diatom, *Haslea ostrearia* maintains a plastidal MEP cycle with a cytosolic MVA pathway and has demonstrated significant crosstalk between these localized elements, suggesting complex regulatory mechanisms perhaps in response to external stimuli and pose potential opportunities to tune both pathways for downstream C5 precursors depending on target terpenoids.

A final distinctive group of phototrophic organisms are purple non-sulfur bacteria, which are identified by a unique color that stems from a combination of pigmented carotenoids. In particular, *Rhodobacter sphaeroides* is a well-established isoprenoid producer, with industrial production of sesquiterpenes valencene and nootkanone demonstrated by BASF (Beekwilder et al., 2014; Schempp et al., 2018). Like many bacteria, *R. sphaeroides* accumulates polyhydroxybutyrate (PHB), a biopolymer with industrial bioplastic applications in of itself, under nitrogen limited conditions. Elimination of the PHB biosynthetic pathway (*phaC*1, *phaC*2) and expression of the heterologous MVA pathway contributed to increased flux through the isoprenoid pathway under nitrogen limited conditions (Orsi et al., 2020b).

As a whole, photosynthetic organisms remain tantalizingly elusive for high titer heterologous isoprenoid production despite advances in “sink engineering” and successes in the production of certain short chain biofuels.

### Formatotrophic Production Pathways

Formate remains an enticing C1 substrate due to the relative ease with which it may be generated. Proposed strategies include the hydration of syngas, the hydrogenation of CO_2_, and electrochemical reduction of CO_2_ using, preferably, renewable generated electricity (Yishai et al., 2016). Bioproduction on formate remains challenging, though recent works have attempted to address this challenge by mapping natural pathways within the context of microbial metabolism (Bar-Even, 2016). The intrinsic nature of formate as an intermediate and availability of natural formate assimilation pathways like the serine, reductive acetyl-CoA, RuMP, XuMP, and reductive glycine pathways have led to the proposal of many synthetic pathways that could theoretically outperform their natural counterparts (Bar-Even, 2016). This hypothesis was encouraged by a previous study that determined formate, not formaldehyde, was the major branch point in *M. extorquens* methylotrophy (Crowther et al., 2008). In particular, this suggested that direct feeding of formate could be energetically beneficial due to the affiliated reduction of NAD^+^ in aldehyde dehydrogenase thereby further supporting formatotrophic pathways (Crowther et al., 2008).

Acting on this hypothesis, *M. extorquens* genes encoding formate-THF ligase, methenyl-THF cyclohydrolase, and methylene-THF dehydrogenase were heterologously expressed in *E. coli* to enable growth on formate through the serine cycle. In combination with downstream modifications, the strain was capable of 90mg/L ethanol production on sugar-free formate minimal medium by adaptive laboratory evolution (ALE; Kim et al., 2019). In a subsequent study, expression of the reductive glycine pathway (rGlyP) in *E. coli* enabled growth on methanol and formate (Kim et al., 2020). Despite clear demonstration of formate-based growth here and *M. extorquens* isoprenoid production on methanol above, few formate derived isoprenoid compounds have been shown. A single exception was a study of the archaea *Methanococcus maripaludis*, which is capable of growth on H_2_, CO_2_, formate, and acetate as substrates under strict anaerobic conditions. Heterologous expression of a geraniol synthase enabled production of 4.0mg/g and 2.8mg/g geraniol on H_2_/CO_2_ and formate feeds, respectively (Lyu et al., 2016). Although meager, this represents a baseline for further isoprenoid production and, with the addition of the groundbreaking production of formatotrophic *E. coli* works, likely represents the first of many formate-based production strains.

So far, we have described a number of routes for isoprenoid production on C1 substrates, including several instances in which whole pathways have been translated between organisms. Life cycle assessment (LCA) and technoeconomic analysis (TEA) will both be critical in quantifying the relative process level sustainability and monetary impacts, validating whether modified microbes are competitive with conventional production on glucose or from petroleum, and prioritizing future optimization opportunities based on projected impact gains. Growth and production on C1 substrates are inherently more sustainable than on pure sugar substrates, however the sustainability of the entire process from cradle-to-gate will be contingent on nontrivial improvement of production titer, rate, and yield. While LCAs and TEAs are common in CO_2_-derived biofuel production, they remain uncommon for all other C1 substrates. Indeed, the first LCA/TEA of a methane-derived bioproduct was only recently published (Fei et al., 2020). Nonetheless this initial study provides a baseline for future valorization of other C1-derived chemicals and, hopefully, represents a first effort to quantify the economic and sustainability advantages of C1 substrates.

## Isoprenoid Production on Lignocellulosic Carbon Sources

Certain microbes are capable of valorizing more complex waste streams due to unique evolutionary predispositions. Here, we describe two strains of oleaginous yeasts, *Yarrowia lipolytica* and *Rhodosporidium toruloides*, capable of high titer isoprenoid production from woody biomass and waste cooking oil (WCO). Lastly, we describe two prototypical isoprenoid production platforms: *P. putida*, which is a prime candidate for conversion of pretreated lignocellulosic biomass, and *B. subtilis*, a candidate bacteria renown for high titer protein production.

### Yarrowia lipolytica

The oleaginous yeast *Yarrowia lipolytica* can naturally assimilate many atypical carbon sources including glycerol, organic acids, succinate, citrate, and even WCO. Likewise, *Y. lipolytica* is of keen interest due to its natural accumulation of β-carotene, farnesene, and linalool. Multi-copy pathway integration has proven especially successful in targeted isoprenoid overproduction (Xie et al., 2015). A recent work applied a random chromosomal integration approach of multiple MVA pathway operons, cofactor modulation, and culture condition tuning produced 25.55g/L α-farnesene on YPD complex medium over 20days in fed-batch production with significant byproduct formation (Liu et al., 2019). A similar strategy led to 6.5g/L production of β-carotene by chromosomal integration of multiple copies of CarB, CarRP, and GGPPS in fed-batch production with over 40g/L lipid byproduct (Larroude et al., 2018). Other reports of note include high squalene production at titers of 531.6mg/L (Gao et al., 2017) and 402.4mg/L (Arnesen et al., 2020). Building upon previous limonene demonstrations with neryl diphosphate synthase (tNPPS1) from *Agastache rugosa* (Korean mint) and limonene synthase from *Solanum lycopersicum* (tomato; Cao et al., 2016), *Y. lipolytica* ultimately yielded 165.3mg/L limonene on glycerol/citrate (Cheng et al., 2019). More comprehensive descriptions of *Y. lipolytica* regulatory changes for production have also been published (Arnesen et al., 2020).

*Y. lipolytica* is also capable of converting fatty acids into C2 substrates through the beta-oxidation pathway and has high native lipid tolerance. Recent works have demonstrated high lipid production of modified *Y. lipolytica* on pretreated lignocellulosic biomass (0.11g lipids/g sugars), even approaching efficiencies observed on glucose (Yook et al., 2020). In fact, *Y. lipolytica* has shown up to 90% DCW lipid accumulation (Park et al., 2018), which demonstrates an encouraging propensity for lipid tolerance. This tolerance has been harnessed by works that have grown *Y. lipolytica* strains on WCO. Growth on WCO increased lipolytic activity (Domínguez et al., 2010) and, in one study, a *Y. lipolytica* strain expressed D-limonene synthase (*Citrus limon*) and L-limonene synthase (*Mentha spicata*) to yield 2.4mg/L of each enantiomer on WCO (Pang et al., 2019). Although this strain has produced only 11mg/L of each enantiomer on complex medium, this stands as an excellent proof of concept for future ALE and optimization studies on WCO.

Xylose catabolism and overcoming catabolite repression are major boundaries to bioproduction on lignocellulosic biomass (Sun et al., 2021). One study showed that carbon source switching enabled production of 20.6mg/L and 15.1mg/L limonene in *Y. lipolytica* from xylose and a 50% lignocellulosic biomass 50% YP rich medium broth, respectively (Yao et al., 2020). This feat was accomplished by overexpression of a native xylulose synthase with heterologous expression of xylitol dehydrogenase and xylulose reductase from *Scheffersomyces stipitis* (Yao et al., 2020). Together, these modifications provided increased G3P production and, ultimately, increased flux through the MVA pathway.

### Rhodosporidium toruloides

*R. toruloides* has attracted attention due to natural high titer lipid and carotenoid accumulation, namely torularhodin, torulene, γ-carotene, and β-carotene, as a convenient carbon storage mechanism under nitrogen-limited conditions (Park et al., 2018). Originally isolated from wood pulp, *R. toruloides* can also metabolize many components of lignocellulosic biomass and has shown simultaneous uptake not only of pentose and hexose sugars, but of p-coumaric acid and aromatic motifs analogous to lignin, which suggest that it could be adapted for direct consumption of lignin (Yaegashi et al., 2017). These traits are further complemented by its ability to thrive on various pretreatment conditions. For example, growth has been demonstrated on ionic liquid (choline α-ketoglutarate) and alkaline pretreated cellulosic biomass, with the latter accumulating 680mg/L α-bisabolene in fed-batch reactor conditions (Yaegashi et al., 2017). Further optimization of the α-bisabolene synthase cassette yielded 4-fold increased titer on lignocellulosic biomass, reaching a final titer of 2.2g/L on corn stover hydrolysate (Kirby et al., 2021). 1,8-cineole was also accumulated to a titer of 1.4g/L on the same substrate, both of which represent titers that, even without significant core metabolic rewiring or downregulation, outstrip comparative *E. coli* and *S. cerevisiae* production. Importantly, pilot scaling of *R. toruloides* to a 1,000L bioreactor for lipid production has been successfully shown (Soccol et al., 2017). Collectively, these traits establish *R. toruloides* as a potential microbial host for lignin valorization. The translation of successful pilot scale *R. toruloides* lipid production platforms to strains with tuned lipid reflex pathways could elevate the yeast to an industrially competitive isoprenoid production platform.

### Pseudomonas putida

As noted with *Y. lipolytica*, tolerance to and simultaneous uptake of multiple carbon substrates is a key phenotype for successful bioproduction on lignocellulosic biomass. The soil bacterium *Pseudomonas putida* maintains significant advantages over common production chassis due to its natural biodegradation pathways and oxidative stress tolerance, which has contributed to its broad proliferation in many environmental niches. Several studies have explored substrate tolerance through toxicity adaptive laboratory evolution (TALE) of *P. putida* (Mohamed et al., 2020; Lim et al., 2021). A recent work integrated three different xylose pathways (Dahms, Isomerase, and Weimberg) on plasmids to enable growth on xylose, a prominent component of degraded hemicellulose (Bator et al., 2019). The combination of pathway expression and ALE resulted in improved tolerance and hence improved growth rate (Bator et al., 2019).

Other production schemes have exploited the natural aromatic tolerance of *P. putida* for growth on substrates like toluene, m-xylene, and p-xylene (Nikel and de Lorenzo, 2018). Comparatively, *P. putida* maintains better *de novo* tolerance toward products that are typically toxic to other organisms (Mi et al., 2014). For example, the saprophytic uptake of organic nutrients and high tolerance to oxidative stress is ideal for biofuel production candidates (Kim and Park, 2014). These traits coupled with overexpression of efflux pumps have shown increased tolerance to short chain C4 and C5 alcohols, which could prove especially valuable for production of isoprenoid biofuels (Basler et al., 2018).

In a hallmark bioproduction study, 2.21g/L of mevalonate were generated by *P. putida* in M9 minimal medium supplemented with 7.5g/L 2,3-butanediol by overexpression of the upper MVA pathway enzymes, namely the native AtoB and the MvaE/MvaS from *Enterococcus faecalis* (Yang et al., 2020). Mevalonate production on 2,3-butanediol proved 6.61- and 8.44-fold higher than production on glucose and glycerol, respectively, though with manageable growth inhibition (Yang et al., 2020). Overall, *P. putida* isoprenoid production has historically been limited to zeaxanthin and geranic acid such that only recently have studies begun addressing MEP/heterologous MVA precursor limitations. One such study exhibited metabolic rerouting of central carbon metabolism from the EMP to ED cycles for better precursor management, namely efficient pyruvate production (Sánchez-Pascuala et al., 2019). This strategy led to a 2-fold increase in carotenoid yield on glucose with plasmid expression of a lycopene synthesis pathway but without any modification to the endogenous MEP pathway (Sánchez-Pascuala et al., 2019).

It is clear that *P. putida* has high innate tolerance to toxic substrates and, as in nature, can adapt to adverse growth conditions. The next, critical stages of realizing *P. putida* as a chemical production platform will be combining advances in ALE, precursor availability, and pathway tuning to enhance terpene synthesis on atypical carbon substrates.

### Bacillus subtilis

*Bacillus subtilis* is one of the best characterized gram-positive bacteria to date and has been an attractive bioproduction candidate due to high titer protein production and high secreting properties. Largely, the industrial focus has been on the production of biologics and enzymes (Pham et al., 2019). *B. subtilis* maintains a faster growth rate than *S. cerevisiae*, a robust metabolism on diverse carbon substrates, and has also shown natural isoprene production at titers comparable to *E. coli* (Zhang et al., 2015). Unlike other chassis organisms like *P. putida*, *B. subtilis* is generally recognized as safe (GRAS), a designation that reduces regulatory boundaries to commercialization. Collectively, these factors suggest that *B. subtilis* could be an excellent candidate for isoprenoid production. Unfortunately, production studies remain relatively limited in part due to a poorly defined metabolic toolkit, which has historically been hampered by a limited subset of selection/counterselection markers that have made genetic manipulation challenging.

Mirroring in *E. coli* from the early 2000s, recent production studies demonstrated that incorporation of amorphadiene synthase (ADS) with overexpression of DXS and IDI led to the accumulation of 20mg/L amorphadiene (Zhou et al., 2013). This titer has dramatically improved to 116mg/L using a CRISPR-cas9 system without culture medium optimization (Song et al., 2021) and then to 416mg/L (Pramastya et al., 2021) with pyruvate supplementation. Another recent study overexpressed the entire MEP pathway excluding IDI, a taxadiene synthase, and a heterologous GGPPS in *B. subtilis*, leading to an accumulation of 17.8mg/L taxadiene (Abdallah et al., 2019). Expression of a squalene synthase from *Bacillus megaterium* also enabled 7.5mg/L production of squalene, which can serve as a precursor to other triterpenoids (Song et al., 2020). Although far from competitive with *E. coli* and *S. cerevisiae*, these initial demonstrations have provided a basis of isoprenoid production in *B. subtilis*. The publication by Song et al. is of particular interest due to their application of CRISPR-cas9 to circumvent boundaries that have historically limited the establishment of *B. subtilis* as an isoprenoid production workhorse. In theory, this approach could be easily translated to the production of other isoprenoid targets.

## Perspectives and Conclusion

The rapid expansion of -omics studies, deep sequencing, and pathway engineering have facilitated bioprospecting of more efficient enzymes, robust combinatorial approaches for tailored isoprenoid production, and the design of altogether novel production pathways. Such tools have also facilitated the exploration of plant derived CYPs and terminal synthases whose subsequent expression has expanded the microbial isoprenoid repertoire to more pharmacologically relevant as well as entirely synthetic terpenoids. In this review, we focused on improvements to isoprenoid precursor biosynthesis and translation of enzymes or pathways between organisms, which could assist in overcoming current major barriers to commercial viability (Zu et al., 2020). Specifically, we highlighted how atypical carbon sources and non-model organisms harbor metabolic advantages that could be harnessed to reduce substrate costs and the associated emissions of bioproduction. Co-substrate utilization by certain organisms as in the case of *R. toruloides* and *P. putida* has the potential to unlock lignocellulosic biomass and many methylotrophs could tap into inexpensive and highly abundant substrates.

We have also described works that capitalized upon the modularity of isoprenoid advances through heterologous expression of entire pathways. Systems engineering strategies are of particular interest for C1 metabolism. The translation of successful whole systems engineering strategies from *E. coli* and *S. cerevisiae* to non-model organisms will prove useful in further optimization. For example, the entire MVA pathway had been expressed in *E. coli* many years ago (Martin et al., 2003) and a decade later, the entire MEP pathway has been expressed in *S. cerevisiae* conversely (Kirby et al., 2016). Both strains have also been extensively mapped through metabolic flux analysis (MFA) which has proven pivotal in metabolic engineering (Orth et al., 2010). The translation of systems engineering strategies like MFA and genome-scale modeling to other organisms will undoubtedly help to inform and improve isoprenoid production in non-model organisms. An MFA of *R. sphaeroides*, for example, showed a mutualistic coupling between its MEP and MVA pathways (Orsi et al., 2020a). Remarkably the true extent of the MEP pathway – MVA pathway relationship could not be resolved as gene knockouts tended to have unpredictable effects on C13 product partitioning but suggested complex regulatory interactions. Nonetheless further work could shed light on how such combined MVA/MEP pathway systems could prove beneficial (Orsi et al., 2020a). Similarly, a metabolic flux reconstruction of *Dunaliella salina* established baseline carbon metabolism during carotenogenesis (Fachet et al., 2020), a critical step in elucidating metabolic bottlenecks. TALE has also proven a powerful strategy for increasing resistance to toxicity of high titer products especially with alcohols. TALE has now been applied to *P. putida* and enhanced toxicity tolerance against the lignocellulosic aromatics such as p-coumaric acid and ferulic acid (Lim et al., 2020; Mohamed et al., 2020). The application of machine learning approaches has enabled extrapolation and gap filling in genome-scale models for rationally designed engineering strategies of non-canonical organisms, as demonstrated to great effect in *Y. lipolytica* (Czajka et al., 2021). And, finally, C1 assimilation pathways have been thoroughly explored, synthetic and natural routes hypothesized (Bar-Even, 2016), then optimal pathways have been heterologously expressed in conventional production chassis (Kim et al., 2020). Having shown adapted growth on C1 substrates there is now a tremendous opportunity to further develop strains for isoprenoid production especially given the comparative sustainability and cost reduction of such substrates with respect to production on refined sugars.

Consortial approaches are also valuable by improving total system productivity. Microbial consortia have proven successful for short chain alcohol production from lignocellulosic biomass (Minty et al., 2013) and have recently been explored in the cross-feeding of methane-derived organic acids produced by *Methylococcus capsulatus* to *E. coli* for the generation of mevalonate at 60mg/L (Lee et al., 2021). Building upon CYP optimization, an *E. coli* and *S. cerevisiae* consortium produced 33mg/L oxygenated taxanes in a consortia where *E. coli* consumes xylose and produces acetate and the precursor taxadiene for consumption and further conjugation in *S. cerevisiae*, respectively (Zhou et al., 2015). Another group produced 0.32g mevalonate/g ethanol in *P. putida* batch experiments (Yang et al., 2019) that, paired with the aforementioned successes in ALE, could provide another promising cross-feeding consortial bioproduction strategy. Finally, isoprenoid production has been expanded to 2,3-butanediol (Yang et al., 2020), which could facilitate consortial bioproduction by subdividing pathways between members.

Exploration of the microbial tree of life has continued to yield an abundant natural diversity of protein homologues, pathway shunts, and mechanisms with which targeted production of isoprenoids has been demonstrably improved. The principal challenge of isoprenoid bioproduction in the next decade will be bridging the knowledge gap between conventional high titer bioproduction on pure sugar substrates and non-model comparatively low titer production on affordable substrates.

## Author Contributions

DC and TSL contributed to the writing of this review and conceived the topic. DC prepared the initial draft, reviewed, and edited the draft. TSL directed the writing process and wrote and reviewed the draft. All authors contributed to the article and approved the submitted version.

## Funding

This work was supported by the DOE Joint BioEnergy Institute (http://www.jbei.org), supported by the US Department of Energy, Office of Science, Office of Biological and Environmental Research, through contract DE-AC0205CH11231 with Lawrence Berkeley National Laboratory.

## Conflict of Interest

The authors declare that they have no known competing financial interests or personal relationships that could have appeared to influence the work reported in this paper.

## Publisher’s Note

All claims expressed in this article are solely those of the authors and do not necessarily represent those of their affiliated organizations, or those of the publisher, the editors and the reviewers. Any product that may be evaluated in this article, or claim that may be made by its manufacturer, is not guaranteed or endorsed by the publisher.
